# Enhanced Physiological and Biochemical Performance of Mung Bean and Maize under Saline and Heavy Metal Stress through Application of Endophytic Fungal Strain SL3 and Exogenous IAA

**DOI:** 10.3390/cells12151960

**Published:** 2023-07-28

**Authors:** Muhammad Aizaz, Ibrahim Khan, Lubna, Sajjad Asaf, Saqib Bilal, Rahmatullah Jan, Abdul Latif Khan, Kyung-Min Kim, Ahmed AL-Harrasi

**Affiliations:** 1 Natural and Medical Science Research Center, University of Nizwa, Nizwa 616, Oman; aizaz@unizwa.edu.om (M.A.); ibrahimkhan@unizwa.edu.om (I.K.); lubnabilal68@gmail.com (L.); sajadasif2000@gmail.com (S.A.);; 2 Department of Applied Biosciences, Kyungpook National University, Daegu 41566, Republic of Korea; rehmatbot@yahoo.com; 3 Department of Engineering Technology, University of Houston, Sugar Land, TX 77479, USA; alkhan@central.uh.edu

**Keywords:** mung bean, maize, endophytes, salinity, heavy metals, sustainable agriculture, oxidative stress

## Abstract

Modern irrigation practices and industrial pollution can contribute to the simultaneous occurrence of salinity and heavy metal contamination in large areas of the world, resulting in significant negative effects on crop productivity and sustainability. This study aimed to investigate the growth-promoting potentials of an important endophytic fungal strain SL3 and to compare its potential with exogenous IAA (indole-3-acetic acid) in the context of salt and heavy metal stress. The strain was assessed for plant growth-promoting traits such as the production of indole-3-acetic acid, gibberellins (GA), and siderophore. We selected two important crops, mung bean and maize, and examined various physiological and biochemical characteristics under 300 mM NaCl and 2.5 mM Pb stress conditions, with and without the application of IAA and SL3. This study’s results demonstrated that both IAA and SL3 positively impacted the growth and development of plants under normal and stressed conditions. In NaCl and Pb-induced stress conditions, the growth of mung bean and maize plants was significantly reduced. However, the application of IAA and SL3 helped to alleviate stress, leading to a significant increase in shoot/root length and weight compared to IAA and SL3 non-treated plants. The results revealed that photosynthetic pigments, accumulation of catalase (CAT), phenolic contents, polyphenol oxidase, and flavanols are higher in the IAA and SL3-treated plants than in the non-inoculated plants. This study’s findings revealed that applying the SL3 fungal strain positively influenced various physiological and biochemical processes in tested plant species under normal and stress conditions of NaCl and Pb. These findings also suggested that SL3 could be a potential replacement for widely used IAA to promote plant growth by improving photosynthetic efficiency, reducing oxidative stress, and enhancing metabolic activities in plants, including mung and maize. Moreover, this study highlights that SL3 has synergistic effects with IAA in enhancing resilience to salt and heavy stress and offers a promising avenue for future agricultural applications in salt and heavy metal-affected regions.

## 1. Introduction

The world’s population is growing at a fast pace, and it is projected that it will reach nearly 10 billion by 2050. This dramatic rise in population is accompanied by an increased proportion of various anthropogenic activities, which lead to the deterioration of plant growth and productivity by imparting abiotic stresses such as salinity and heavy metals [1]. In order to sustain this population, food production needs to increase by 60–100% from the current level [2]. So far, many attempts have been made to increase crop tolerance against various stresses, including high salt and heavy metals, through applications of different chemicals, conventional breeding, and plant omics technologies [3]. However, such approaches are not always feasible and might create long-term negative impacts on the ecosystem. Therefore, cost-effective and eco-friendly strategies should be devised to cope with various stressors and improve sustainable agriculture [4]. During the past two decades, endophytes have been used as a valuable source to improve plant growth and adaptation to adverse stress conditions [5,6]. Endophytes are synergistic microorganisms, colonizing intracellularly and enhancing host plant growth by improving nutrient uptake and tolerance to environmental stresses [7]. The plant growth promoting endophytes (PGPE) mediated stress alleviation in the host through two mechanisms, activation of host response systems and biosynthesis of anti-stress compounds such as enzymatic and non-enzymatic antioxidants and phytohormones [8]. Several endophytic fungal and bacterial strains have been examined for their potential to promote plant growth in stress conditions [9,10].

Similarly, the exogenous application of phytohormones has attained a substantial interest in alleviating the adverse effects of salt and heavy metals stress [11]. Exogenous treatment of plants with phytohormones is an effective strategy for protecting plants against stress-induced toxic effects. The indole-3-acetic acid (IAA) is a small signaling molecule that considerably supports plant growth and development and acts virtually, to some extent, to increase plant adaptation to toxic effects of stresses [12]. It has been shown that IAA plays crucial roles in various aspects of plant growth and development, including responses to environmental stresses [13].

The endophytic fungal strain *Fusarium proliferatum* SL3 and IAA was examined for its phytostimulatory characteristics and salt stress alleviation potentials in the current study. We assumed that the selected endophyte and IAA treatment might be useful to confer fitness benefits to host plants through various morphological and architectural changes and activations of biochemical and metabolic pathways. To verify this hypothesis, we conducted the current study on two important crops, mung bean and maize, under various levels of salt and heavy metals stresses.

Mung bean (*Vigna radiata* L.) is an important pulse crop, possessing high nutritive value. It is widely cultivated in tropical and subtropical climates all around the world [14]. Mung bean is an ideal pulse crop for physiological studies due to its short life cycle and growth in a wide range of soil and environments [15]. Maize has also become one of the most extensively utilized model plant species due to its tremendous phenotypic and genotypic diversity. Mung beans have been used as an integral part of the human diet for low-fat and high-protein contents with prebiotic potential against metabolic syndromes such as diabetes and cardiovascular diseases. In addition to proteins, mung beans are also rich in vitamins, fibers, minerals, and a significant amount of divalent cations, such as calcium, magnesium, iron, and zinc [16]. Maize (*Zea mays* L.) is considered the third most important cereal crop after wheat and rice, mainly utilized as feed, food, and raw material for diverse industrial applications [17]. Maize is cultivated virtually all over the world from arid and semi-arid to humid and semi-humid areas due to its agricultural, nutritional, and socioeconomic importance [18]. It has been reported that salinity increases heavy metal mobilization in soils; this would be an important additional adverse phenomenon [19]. Looking at the increasingly detrimental effects of salinity and heavy metals, the application of endophytes and organic phytohormones represents a promising and sustainable approach to address the challenges faced in modern agriculture and environmental sustainability. These microbes and phytohormones can contribute to improved plant growth, nutrient uptake, and stress tolerance, ultimately enhancing the growth and productivity of crops such as mung bean and maize [20].

Overall, the current study aims to investigate the responses of mung bean and maize plants to salt and heavy metal stresses and assess the potential of an endophytic fungal strain SL3 and a phytohormone IAA in mitigating the detrimental effects of these stressors. The findings from such research endeavors can provide valuable insights into developing sustainable agricultural practices that improve crop productivity while minimizing the negative impacts on the environment.

## 2. Materials and Methods 

### 2.1. Isolation and Screening of SL3 Fungal Strain

The endophytic fungal SL3 strain was isolated from plants using our lab-established protocols [5]. To assess the tolerance of the SL3 fungal strain to salt and heavy metal stress, we tried to grow the SL3 fungal strain on PDA media with different concentrations of NaCl (100–400 mM) and Pb (1–3) mM and concluded that the strain can tolerate stress up to 300 mM NaCl and 2.5 mM Pb. To further validate our finding and to study the beneficial symbiotic relationship of SL3 with different plants, we conducted our experiments on the two important crops, maize and mung. The strain was also tested for its ability to produce indole acetic acid (IAA) using the technique described by Gang and coworkers [21].

### 2.2. Identification and Phylogenetic Analysis of SL3

SL3 mycelia were ground in liquid nitrogen with a mortar and pestle, and genomic DNA (gDNA) was isolated using a DNeasy plant mini kit (QIAGEN, Valencia, CA, USA). PCR products of the internal transcriber region (ITS) of the gDNA samples were purified and sequenced commercially from Macrojen (Seoul, Republic of Korea). For amplification and sequencing of the ITS region, the same universal primer pairs of ITS4 (5′-TCCTCCGCTTATTGATATGC-3′) and ITS5 (5′-TCCGTAGGTGAACCTGCGG-3′) were used. BLAST algorithm, in comparison to sequences in the NCBI database (http://www.ncbi.nlm.nih.gov/ (accessed on 15 May 2023)), was used to determine the homology of different nucleotide sequences of the selected isolates. MEGA (version 11.0) software with 1000 bootstrap replicates was used to align closely related sequences by the Clustal-W method. The neighbor-joining method was used to perform phylogenetic inference. The validity of the trees and the accuracy of the interior branches were tested using bootstrapping. The sequenced data were submitted in gene bank accessions (*Fusarium proliferatum* strain SL3 (OR143781; https://submit.ncbi.nlm.nih.gov/subs/?search=SUB13557924 (accessed on 15 May 2023))).

### 2.3. Determination of IAA by GC/MS

The culture filtrate (CF) of SL3 was examined for the presence of IAA by GC/MS, as described by [22]. In this experiment, the SL3 isolate was grown in 50 mL PDB medium and incubated for 7 days at 28 °C in a shaking incubator at 120 rpm. The culture was filtered to separate the CF, which was then acidified (pH 2.8) by adding 1 N HCl, and 40 μg mL^–1^ [D5]-IAA was added as the IAA internal standard. An equivalent volume of ethyl acetate was used to extract the acidified CF, and the aliquot was completely evaporated using a rotatory evaporator. According to [23], the acidified CF was quantified using a GC–MS/SIM system (Agilent Technologies, Palo Alto, CA, USA; 6890 N network GC system and 5973 network mass selective detector).

### 2.4. Determination of Siderophore Production

The SL3 strain was cultured on CAS agar medium to examine its siderophore activity [24]. An amount of 60.5 mg of chromeazurol S was dissolved in 50 mL of distilled water (DW) to prepare 100 mL of CAS indicator solution. Then, this solution was mixed with 10 mL of iron III solution. [25,26]. An amount of 100 mL of basal agar media was prepared by mixing 0.01 g of NH_4_Cl, 0.03 g KH_2_PO_4_, 0.5 g of L-asparagine, 0.05 g of NaCl, and 3 g of 3-(N-morpholino) propane sulfonic acid (MOPS) in 83 mL of DW. After that, the solution was added to 1.5 g agar, 2 mL of the 50% glucose solution, and 10 mL of CAS indicator in a sterilized condition. The formation of the orange zone around the fungus was considered a positive indication of siderophore production.

### 2.5. Extraction and Quantification of Gibberellins (GAs) in Culture Broth

The protocol established by Lee and Foster [27] was used for the extraction and quantification of the GAs. After 7 days of incubation, the CF was fractionated using preparative high-performance liquid chromatography (HPLC) equipped with a reverse-phase C18 column. The GA-containing fractions were collected and further analyzed using gas chromatography–mass spectrometry (GC–MS) with the selected ion monitoring (SIM) option. After the GC–MS analysis, all the data were collected and analyzed. The three major ions of both the supplemented [2H_2_] GA internal standard and the GAs present in the sample were monitored simultaneously. In order to quantify GA, the Kovats retention index (KRI) value was calculated by comparing the retention times and peak ratios between the denatured and non-denatured GAs. To determine the retention time, the hydrocarbon standards were used to calculate the Kovats retention index (KRI) value, while GAs were quantified by comparing the peak area ratios between non-deuterated and deuterated GAs [28]. Each sample was replicated five times, and the experiment was repeated thrice to ensure reproducibility and validate the results.

### 2.6. Seeds Surface Sterilization and Germination

Mung and maize seeds were obtained from Agriculture Research Center KP, Pakistan. After being washed with distilled water, the seeds were surface-sterilized by immersing in 70% ethanol (*v*/*v*) and 2.5% sodium hypochlorite (*w*/*v*), followed by washing three times with autoclaved distilled water. The surface sterilized seeds were carefully transferred to petri dishes with the addition of 5 mL dH_2_O under hygienic conditions. After 3 days, the germinated seeds were carefully transferred to 10 × 9-cm plastic pots filled with horticulture substrate containing coco peat moss (10–15%), coco peat (45–50%), pertile (35–40), NH^+^ (ca.0.09 mg/g), KO (c.0.1 mg/g), zeolite (6-8%) with NO_3_ (ca. 0.205 mg/g), and PO (ca.0.35 mg/g). The pots were placed in a growth chamber at 28°C temperature, 8/16 h night/day photoperiod, and 65 ± 3% relative humidity.

### 2.7. Experimental Design

After five days, the seedlings of both crops were exposed to 13 different growth conditions: (a) control plants (distilled water); (b) plants inoculated with SL3; (c) plants treated with 50 ppm IAA; (d) plants with 50 ppm IAA+SL3; (e) plants with 2.5 mM Pb; (f), plants with 2.5 mM Pb+SL3; (g) plants with 2.5 mM Pb + 50 ppm IAA; (h) plants with 300 mM NaCl; (i) plants with 300 mM NaCl+SL3; (j) plants with 300 mM NaCl + 50 ppm IAA; (k) plants with 2.5 mM Pb + 300 mM NaCl; (l) plants with 2.5 mM Pb + 300 mM NaCl+SL3; (m) plants with 2.5 mM Pb+ 300 NaCl + 50 ppm IAA.

### 2.8. Assessment of Growth Attributes

Plants were irrigated with 300 mM NaCl and treated with endophytic fungal strain (SL3) within 2-day intervals for 1 month. The experiment was performed with 5 replicates per treatment. Plants were harvested after one month, and growth attributes (root/shoot length) and biomass (root/shoot weight) were measured. The plant materials were immediately frozen in liquid nitrogen and stored at −80 °C until further analysis.

### 2.9. Assessment of Photosynthetic Pigments

Frozen leave samples from each group were taken and crushed immediately with liquid nitrogen. An amount of 1 mL of 80% acetone was added to 200 mg of each sample and homogenized with vortex. The homogenate was centrifuged at 10,000 rpm for 5 min, and 100 µL supernatant from each sample was transferred into a fresh 96-well plate. The concentration of chlorophyll-a, chlorophyll-b, and carotenoids was determined using a spectrophotometer at an absorbance of 663 nm, 645 nm, and 470 nm wavelength, respectively. The concentration of the photosynthetic pigments was calculated using the extension coefficients and equation given in Barnes’s method [29], with slight modifications.

### 2.10. Assessment of Total Protein Contents

The analytical spectroscopic method of Bradford [30], with slight modifications, was used to determine total protein contents. Finely crushed leaf samples from each group were added to 1 mL extraction buffer formulated by Arulsekar and Parfitt [31] and mixed gently. The homogenate samples were centrifuged at 4000 rpm for 10 min at 2 °C. Supernatants were transferred into fresh 1.5 mL tubes and added with 150 µL of Bradford reagent. After a five-minute incubation at room temperature, a 150 µL sample from each treatment group was pipetted into a 96-wells microplate and measured the absorbance at a wavelength of 595 nm using an xMark™ microplate reader.

### 2.11. Assessment of Catalase Activity

Fresh leaves (200 mg) were crushed into fine powder in liquid nitrogen and added to 1 mL of extraction buffer (50mM Tris HCl (pH 7.0), 10% glycerol, 3 mM MgCl2, 1 mM EDTA, and 1% pvp). The mixture was centrifuged at 10,000 rpm at 4 °C for 15 min. An amount of 240 µL supernatant was collected and mixed immediately with an equal volume of 0.1 mM phosphate buffer (pH 7.0) and 120 µL of 0.2 M H_2_O_2_. The optical density (OD) was measured at 240 nm using a spectrophotometer.

### 2.12. Assessment of Total Phenolic Content

The Folin–Ciocalteu method [32], with slight changes, was used to determine the total phenolic content of both mung and maize samples. A total of 5 g plant material was crushed finely in liquid nitrogen, and 150 µL extract from each sample was gently mixed with 1.5 mL of 2% Na_2_CO_3_ using vertex. Then, 0.1 mL of CuSO_4_ and 0.1 mL of sodium and potassium tartrate were added, and the mixture was kept at room temperature for 4 min, followed by the addition of 0.4 mL of 0.5 M sodium hydroxide. The mixture was centrifuged at room temperature for 10 min at a speed of 10,000 rpm. The collected supernatant was incubated in the dark for 30 min, and its absorbance was measured at 750 nm using an xMark™ microplate reader. For external calibration of the curve, ethanolic solutions with different concentrations of gallic acid, i.e., 0.00, 0.25, 0.50, 0.75, and 1 mM, were prepared.

### 2.13. Assessment of Polyphenol Oxidase

The polyphenol oxidase (PPO) assay was carried out using the method described by Illera et al. [33]. In short, 100 µL of sample from each treatment group was mixed with 2.9 mL of substrate solution (0.05 M catechol solution prepared in a 0.1 M phosphate buffer having pH 6.5). The resultant mixture was kept at 30 °C in a water bath. Oxidation of catechol was determined spectrophotometrically at 420 nm. The solution of catechol was used as a blank.

### 2.14. Assessment of Flavanol Contents

To extract and asses total flavanol contents, Park’s method [34] was utilized. Fresh leaf samples were ground finely in liquid nitrogen with a mortar and pestle. Then, 0.5 g powder was mixed with 1 mL of 80% methanol and kept at room temperature for 24 h. The mixture was then centrifuged for 15 min at 10,000 rpm, and the resultant supernatant was collected and combined with an equal amount of 2% aluminum chloride diluted in 95% ethanol. After 20 min incubation at room temperature, absorbance was measured at 390 nm spectrophotometrically.

### 2.15. Statistical Analysis

All experiments were carried out in five biological and three technical replications, and the resultant data were combined for interpretation. One-way ANOVA based on DMRT for inter and intra-treatments was carried out, and Duncan’s multiple range test (DMRT) was used to measure specific differences between pairs of means. A completely random design was applied to compare the mean values of the various treatments. Pearson’s correlation coefficient was used to determine variance significance in various morpho-physiological, biochemical, and antioxidative traits under normal, Pb, and NaCl-stressed, IAA-treated, SL3-inoculated, and non-inoculated conditions.

## 3. Results

### 3.1. Assessment of SL3 Tolerance to NaCl and Pb

**Figure 1:**
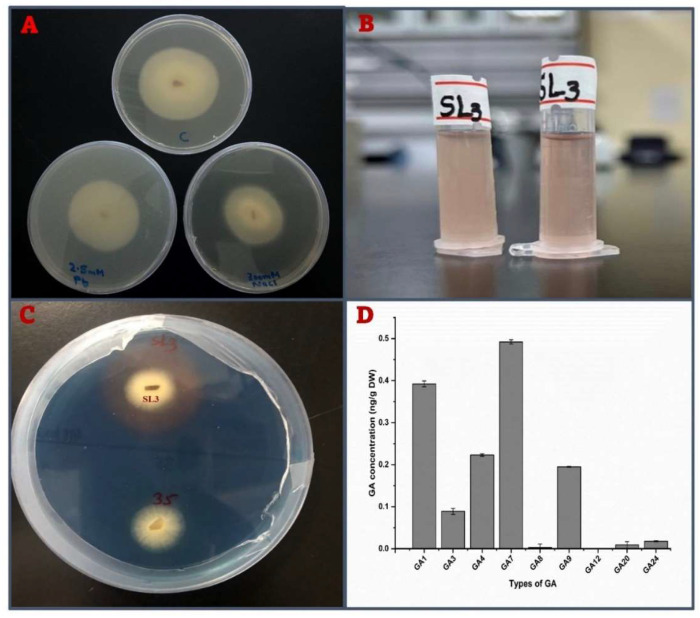
(**A**) *Fusarium proliferatum* SL3 growth on PDA medium under control, 2.5 mM Pb, and 300 mM NaCl stress conditions. (**B**) Indole-3-acetic acid (IAA) production by SL3. (**C**) Siderophore production by SL3. (**D**) Gibberellin (GA) production by SL3.

PDA media plates were prepared with 300 mM NaCl and 2.5 mM Pb to test the growth ability of SL3 under high salt (NaCl) and heavy metal (Pb) stress conditions. The results revealed that SL3 can grow on the media supplemented with 300 mM NaCl and 2.5 mM Pb. However, its growth was significantly reduced compared to the control condition (distilled water) (Figure 1A). This reduction in growth suggests that SL3 is capable of surviving and growing to some extent under high NaCl and Pb concentrations.

### 3.2. Quantitation of IAA in the Fungal Culture

Determination of IAA in the SL3 culture filtrate was carried out by using GC–MS. SL3 produced a significant amount of IAA, i.e., 1.32 ± 0.07µg/mL. Quantitative analysis was very much in agreement with the result drawn from the qualitative analysis as SL3 (Figure 1B).

### 3.3. Siderophore Production by SL3

The results demonstrated that the SL3 strain was capable of producing siderophores on CAS agar medium [24]. The orange zone was observed around the fungal isolates, indicating the interaction between the siderophores produced by the SL3 strain and the iron present in the medium [35]. After three days of incubation at 25 °C, clear zones were also observed around the growing fungal colonies demonstrating significant siderophore activity of the SL3 strain (Figure 1C).

### 3.4. Determination of Gibberellins in SL3 Culture Filtrate

The SL3 isolate was grown in broth media for 10 days on a rotary shaker, and its CF was tested for the presence of GAs using GC–MS/SIM. The analysis revealed that the SL3 culture filtrate contained both the biologically active and inactive forms of GAs. Nine types of biologically active Gas, including GA_1_, GA_3_, GA_4_, GA_7_, GA_8_, GA_9_, GA_12_, GA_20_, and GA_24_, were found in the SL3 culture filtrate. It was observed that the SL3 strain released a particularly high quantity of GA_7_, i.e., 0.492 ± 0.005 ng/mL in the culture media. The inactive types of GA present in the culture filtrate of the SL3 isolate were GA8, GA12, GA20, and GA24 (Figure 1D).

### 3.5. Fungal Strain Identification and Phylogenetic Analysis

**Figure 2:**
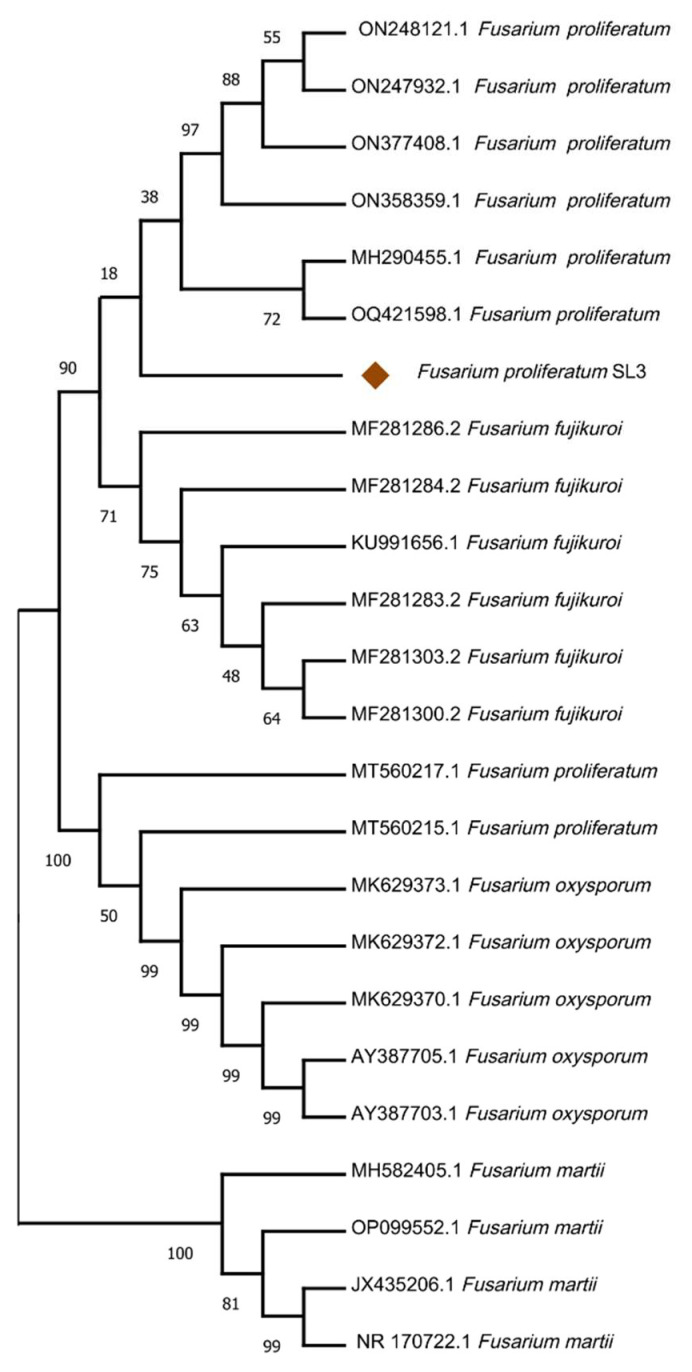
The phylogenetic tree of *Fusarium proliferatum* SL3 strain constructed by the neighbor-joining method using the MEGA-11 software with 10,000 bootstrap replicates.

To infer the phylogenetic position of the SL3 strain, the sequenced ITS region was compared to the sequences in the NCBI database through BLAST search analysis (http://www.ncbi.nlm.nih.gov/ (accessed on 15 May 2023). The results revealed that the SL3 exhibited a higher level of similarity with *Fusarium proliferatum* (Figure 2).

### 3.6. Effect of SL3 Inoculation and IAA Treatment on Growth Attributes under NaCl and Pb Stress

**Figure 3:**
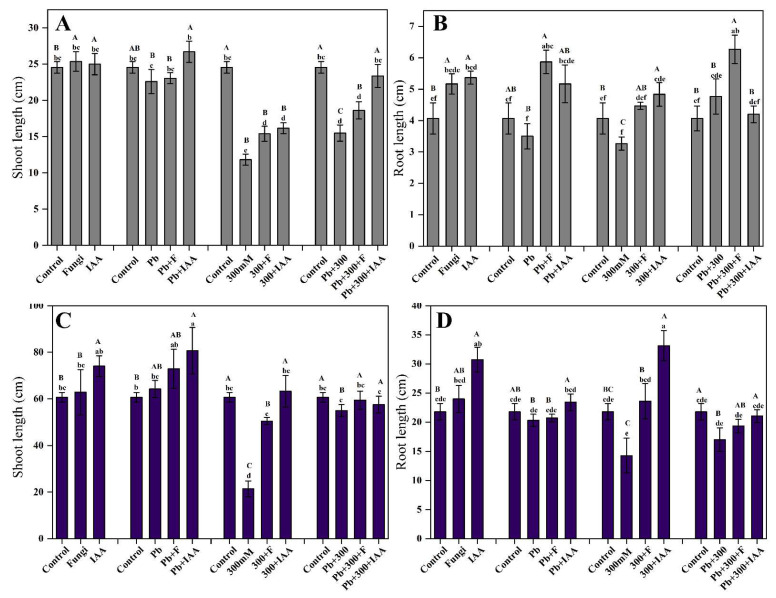
Effect of inoculation of SL3 fungal strain on growth of mung and maize plants under control, 2.5 mM Pb, and 300 mM NaCl stress conditions. (**A**) Mung shoot length. (**B**) Mung root length. (**C**) Maize shoot length. (**D**) Maize root length. Bars with different capital letters indicate significant differences (*p* ≤ 0.05) among all treatments based on DMRT. Bars with different small letters indicate significant differences (*p* ≤ 0.05) among control, stress, fungal-inoculated, and exogenous IAA treatments based on DMRT.

Our results revealed that in control conditions, treatment of the endophytic fungal strain (SL3) promotes a greater shoot length in mung plants by 3.2% compared to their non-inoculated counterparts. The Pb stress slightly affects plant growth in terms of shoot length, while 300 mM NaCl stress significantly inhibited plant shoot length. NaCl stress decreased shoot length by 51.8% compared to control plants (distilled water added), while the endophytic fungal inoculation and IAA treatment significantly alleviated the salt stress and enhanced shoot length by 30.1% and 36.6%, respectively. Similarly, the negative effect of Pb stress was also alleviated in mung plants inoculated with SL3 endophytic fungal strain and treated with IAA. Surprisingly, the 50 ppm IAA treatment considerably mitigated the stress effects of both 300 mM NaCl and 2.5 mM Pb and promoted shoot length by 50.7% compared to their non-treated counterparts (Figure 3A). Similar trends were observed in maize plants as in control conditions; treatment of SL3 and IAA enhanced maize plant shoot length by 3.5% and 22%, respectively. Unexpectedly, Pb stress has a positive effect on shoot length in maize plants compared to their non-stressed counterparts. Overall, our findings showed that IAA treatment was more effective in mitigating stresses imposed by NaCl and Pb compared to SL3 endophytic fungal strain (Figure 3C).

The root length of both mung and maize plants was also significantly greater in fungal and IAA-exposed plants than in control ones. The results revealed that the SL3 strain significantly enhanced root length compared to IAA treatment both in NaCl and Pb stress. The results showed that SL3 promoted the root length of Pb and NaCl-stressed mung plants by 67.7% and 36.7%, respectively, compared to their respective controls. Importantly, SL3 inoculation mitigated the combined stress of NaCl and Pb by promoting the root length of mung plants up to 31.4% compared to their non-inoculated counterparts (Figure 3B). In non-stressed conditions, IAA treatment promotes the root length of maize plants by 41.1% compared to IAA non-treated plants. However, when a combined treatment of SL3 and IAA is applied, root length is increased by up to 29% compared to the root length of control plants. Our findings demonstrated that the 300 mM NaCl stress had a greater impact on maize root length than the Pb stress. However, both SL3 and IAA treatments are effective in mitigating the negative effects of salt stress. For instance, maize plants treated with 300 mM NaCl and 50 ppm IAA have the longest roots, i.e., 132% longer than their IAA non-treated stressed counterparts (Figure 3D).

**Figure 4:**
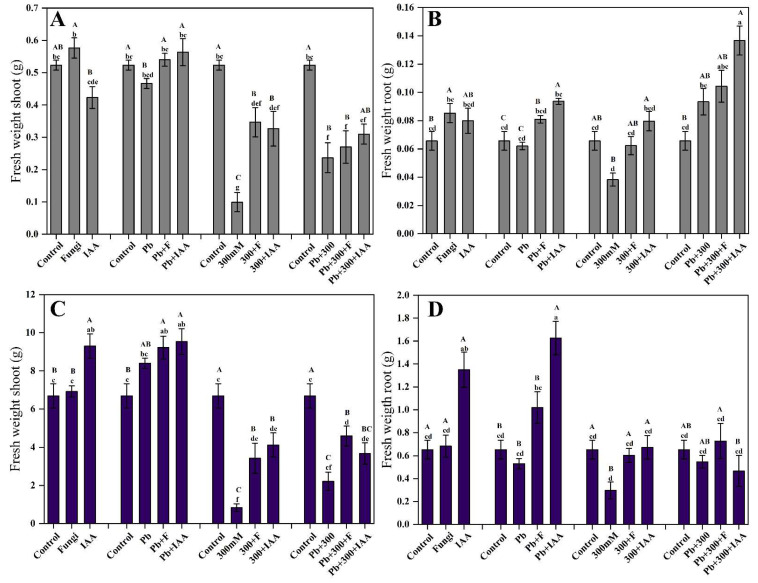
Effect of inoculation of SL3 fungal strain on biomass of mung and maize plants under control, 2.5 mM Pb, and 300 mM NaCl stress conditions. (**A**) Mung shoot weight. (**B**) Mung root weight. (**C**) Maize shoot weight. (**D**) Maize root weight. Bars with different capital letters indicate significant differences (*p* ≤ 0.05) among all treatments based on DMRT. Bars with different small letters indicate significant differences (*p* ≤ 0.05) among control, stress, fungal-inoculated, and exogenous IAA treatments based on DMRT.

Results showed that both mung and maize plants inoculated with SL3 and treated with IAA increased plant biomass significantly compared to non-inoculated plants in both normal and stressed conditions. In mung plants, the individual treatment of SL3 and IAA results in a 10.2% increase and 19.1% decrease, respectively, in shoot weight compared to the control plants. However, when both SL3 and IAA are applied together, then they show a synergistic effect, and the shoot weight is significantly increased by 36.9% compared to the control plants. Similar to root/shoot length, Pb stress has no significant negative effect on root/shoot weight, and salt stress reduces the root/shoot biomass significantly. However, endophytic SL3 strain and IAA treatment act antagonistically to the inhibitory effect of salt and heavy metal stresses and significantly improve root/shoot weight both in mung and maize plants (Figure 4A–D).

### 3.7. Effects of SL3 and IAA Treatment on Photosynthetic Pigments of Mung Bean and Maize under NaCl and Pb Stress

**Figure 5:**
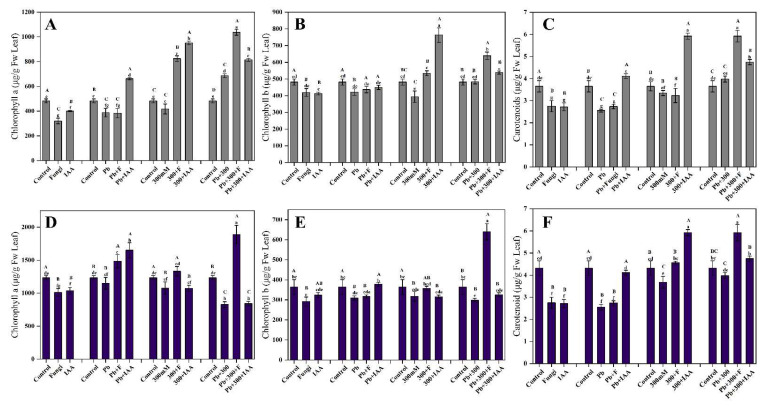
Effect of inoculation of the SL3 fungal strain on photosynthetic pigments. (**A**) Mung Chlorophyll-a. (**B**) Mung Chlorophyll-b. (**C**) Mung Carotenoids. (**D**) Maize Chlorophyll-a. (**E**) Maize Chlorophyll-b. (**F**) Maize Carotenoids under control, 2.5 mM Pb, and 300 mM NaCl stress conditions. Bars with different capital letters indicate significant differences (*p* ≤ 0.05) among all treatments based on DMRT. Bars with different small letters indicate significant differences (*p* ≤ 0.05) among control, stress, fungal-inoculated, and exogenous IAA treatments based on DMRT.

Results shown in Figure 5A–C illustrated that in control conditions, treatment of mung plants with IAA and SL3 reduced the contents of photosynthetic pigments compared to their non-treated counterparts. Under Pb stress, both IAA and SL3 treatments slightly improved the contents of the photosynthetic pigments in mung plants. It is interesting to note that mung plants inoculated with SL3 and treated with 2.5 mM Pb stress showed higher concentrations of chlorophyll-b and carotenoids compared to their control counterparts. In NaCl stress conditions, both IAA and SL3 treatments significantly improved the contents of all the photosynthetic pigments. Our results showed that the highest chlorophyll-a and carotenoids were found in SL3-inoculated mung plants, which were exposed to simultaneous stress of NaCl and Pb. The results obtained for maize were consistent with those obtained for mung bean in terms of the effects of IAA and SL3 treatment on photosynthetic pigments under salt and heavy metal stress conditions. As in control conditions, treatments of IAA and SL3 decreased the concentration of the photosynthetic pigments significantly, and SL3 inoculation remarkably increased the contents under the combined stress of NaCl and Pb. Moreover, it is inferred that individual, as well as combined treatments of IAA and SL3, improved the contents of photosynthetic pigments under salt and heavy metal stress conditions in maize plants (Figure 5D–F).

### 3.8. Effect of SL3 and IAA Treatments on Total Protein Contents of Mung Bean and Maize under NaCl and Pb Stress

**Figure 6:**
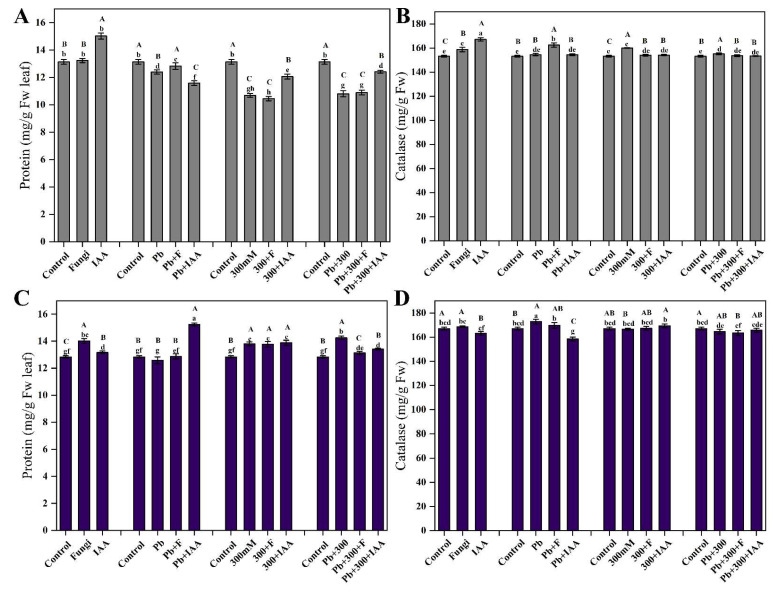
Effect of inoculation of the SL3 fungal strain on total protein and catalase contents. (**A**) Mung total protein contents. (**B**) Mung total catalase (CAT). (**C**) Maize total protein contents. (**D**) Maize total catalase starch. Bars with different capital letters indicate significant differences (*p* ≤ 0.05) among all treatments based on DMRT. Bars with different small letters indicate significant differences (*p* ≤ 0.05) among control, stress, fungal-inoculated, and exogenous IAA treatments based on DMRT.

Our results showed that in control conditions, IAA and SL3 treatment slightly increased total protein contents in mung bean and maize plants, respectively. SL3 inoculation increased while IAA treatment decreased the total protein contents in mung plants under Pb stress conditions. Under salt stress conditions, both IAA and SL3 treatments significantly reduced the total protein contents compared to control plants. In combined stress conditions of 300 mM NaCl and 2.5 mM Pb, SL3 inoculation had no significant effect; however, IAA significantly enhanced the total protein content (Figure 6A). The results revealed that IAA treatment significantly increased the total protein contents in maize plants under individual Pb and combined Pb+300 mM NaCl stress conditions. An increase was recorded in total protein contents under salt stress conditions both in inoculated and non-inoculated maize plants compared to control ones (Figure 6C).

### 3.9. Effect of SL3 and IAA Treatments on Catalase Activity of Mung Bean and Maize under NaCl and Pb Stress

Our results imply that under control as well as NaCl and Pb stress conditions, the catalase activity in both IAA and SL3-treated and non-treated plants was not altered significantly. The obtained results suggest that IAA and SL3 did not influence the ability of the mung and maize plants to mitigate the oxidative stress caused by NaCl and Pb treatments (Figure 6B,D).

### 3.10. Effect of SL3 and IAA Treatments on total Phenolic Contents of Mung Bean and Maize under NaCl and Pb Stress

**Figure 7:**
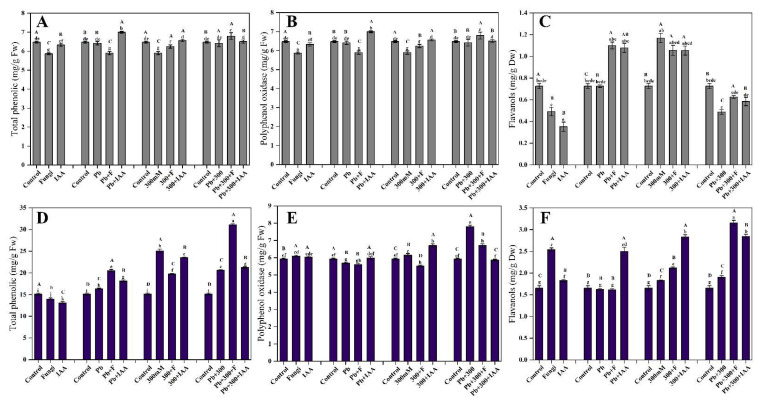
Effect of inoculation of the SL3 fungal strain on reactive species production and different antioxidants. (**A**) Mung phenolic contents. (**B**) Mung polyphenol oxidase. (**C**) Mung flavanols. (**D**) Maize phenolic contents. (**E**) Maize polyphenol oxidase. (**F**) Maize flavanols. Bars with different capital letters indicate significant differences (*p* ≤ 0.05) among all treatments based on DMRT. Bars with different small letters indicate significant differences (*p* ≤ 0.05) among control, stress, fungal-inoculated, and exogenous IAA treatments based on DMRT.

The total phenolic contents were analyzed in both IAA and SL3-treated and untreated mung and maize plants under control and stress conditions of NaCl and Pb. The phenolic content of mung plants tends to decrease under control and Pb stress conditions, whereas increases in phenolic content were observed in mung plants inoculated with SL3 under NaCl and Pb+NaCl stress conditions. In control conditions, a reduction in total phenolic contents was also observed when maize plants were treated with IAA and SL3. The total phenolic contents were significantly enhanced in stress conditions of NaCl and Pb compared to control maize plants. In 300 NaCl stress, both IAA and SL3 tend to decrease the phenolic contents compared to their stressed counterparts. The highest phenolic contents were recorded in SL3 inoculated maize plants treated with combined stress of NaCl and Pb (Figure 7A,D).

### 3.11. Effect of SL3 and IAA Treatments on Polyphenol Oxidase Content of Mung Bean and Maize under NaCl and Pb Stress

SL3 inoculation caused a decrease in PPO levels in both control and stressed plants of mung bean and maize. On the other hand, IAA treatment increased PPO levels under Pb and NaCl-induced stress conditions. The highest PPO level was observed in maize plants that were subjected to the combined stress of Pb and NaCl (Figure 7B,E).

### 3.12. Effects of SL3 and IAA Treatment on Flavanols Content of Mung Bean and Maize under NaCl and Pb Stress

The results revealed that in mung plants, both IAA and SL3 treatments led to a significant decrease in flavanol contents drastically decreased the flavanols contents. However, when the mung plants were exposed to stress conditions induced by Pb and NaCl, the treatment of both IAA and SL3 significantly enhanced the flavanol contents. Interestingly, when the mung plants were exposed to the combined stress of Pb and NaCl, the flavanol contents decreased significantly. However, the application of IAA and SL3 showed restorative effects to some extent. The highest flavanol contents were observed in mung plants that were treated with 300 mM NaCl stress, both under IAA and SL3-treated and untreated conditions. This suggests that the application of NaCl stress, along with IAA or SL3 treatment, resulted in a higher accumulation of flavanols in the mung plants (Figure 7C). As shown in Figure 7F, SL3 inoculation significantly increased the flavanol contents in maize plants both under control and stress conditions, except in Pb-induced stress conditions where SL3 did not show a significant effect on flavanol contents. A significant increase was recorded in flavanol contents both under Pb and NaCl-induced stress conditions, which was further enhanced with the application of IAA and SL3.

## 4. Discussion

Salinity stress refers to the elevated levels of different salts, particularly NaCl, in the soil or water and all the associated problems, including a reduction in the growth and productivity of plants. When plants are exposed to high salinity, it disrupts their normal physiological processes [36]. The excess salts can cause osmotic stress [37], leading to water imbalance and dehydration in plants [38]. This can result in reduced water uptake, inhibition of nutrient absorption, and overall stunted growth [1,39]. Additionally, salt stress can induce ion toxicity, as excessive sodium and chloride ions accumulate in plant tissues, interfering with essential metabolic processes [40]. Similarly, heavy metals present in the environment, such as lead [41,42], cadmium, mercury, and arsenic, pose a significant threat to plant health [43,44]. These metals can accumulate in the soil through various industrial activities, pollution, or natural processes. When plants are exposed to high concentrations of heavy metals, they can interfere with essential plant functions. Heavy metals can disrupt enzymatic activity, inhibit photosynthesis, and generate reactive oxygen species (ROS), leading to oxidative stress and damage to cellular structures [43,45]. Traditional approaches, such as using agrochemicals, and modern molecular breeding techniques, such as genome editing technologies, have been employed to develop crop varieties with salinity tolerance [2,46]. However, these approaches may not always be feasible and can potentially have serious implications for the ecosystem [1]. In this context, endophytic microbes play a crucial role in enhancing salt and heavy metal stress resistance [47]. These microbes reside within plant tissues symbiotically and help restore ion homeostasis and alleviate the negative consequences of ion toxicity and oxidative stress caused by salt and heavy metal-induced stress [5,48]. The exogenous application of IAA has also been reported to alleviate the adverse effects of salt and heavy metal stress. It has been shown that IAA treatment enhances the tolerance of plants to high salinity by regulating ion homeostasis, improving antioxidant defense mechanisms, and reducing oxidative damages that eventually lead to alleviating stress conditions [49,50]. Additionally, IAA treatment can promote root growth and development, which can be beneficial for plants to explore a larger soil volume and access water and nutrients more efficiently [51].

The current study aimed to explore the beneficial effects of the SL3 endophytic strain and a growth regulator, IAA, on NaCl and Pb stress mitigation in mung and maize plants. The SL3 strain was screened for its ability to produce IAA, GA, and siderophore in vitro on chemically defined media. We also confirmed the NaCl and Pb stress resistance in the SL3 strain by culturing on PDB media supplemented with 300 mM NaCl and 2.5 mM Pb concentration. Our findings have demonstrated that mung and maize plants treated with SL3 and IAA exhibited improved growth attributes such as shoot/root length and weight compared to non-treated plants by mitigating the detrimental effects of NaCl and Pb stress. Similar results were found in previous studies that endophytic microbes and an exogenous supply of IAA mitigate the negative effects of salt and heavy metal stress on plants and enhance their growth potential under such conditions [52,53,54,55]. The obtained results are in agreement with previous studies [51,56,57] that application of endophytic microbes and exogenous IAA has a positive effect on the photosynthetic pigments of the plants leading to an increase in their contents. These results suggest that IAA and SL3 increased the chlorophyll and carotenoid contents in the treated plants, possibly by alleviating ion toxicity [58,59,60], reducing oxidative stress [61], and contributing to increased chloroplast metabolism [62]. In plants, salt and heavy metal-induced stress led to the overgeneration of oxygen radicals and their derivates called reactive oxygen species (ROS) [63]. Excessive cellular levels of ROS can cause direct injury to various cellular components, including nucleic acids, proteins, lipids, and other organelles [64,65]. This oxidative damage can ultimately lead to cell death [66,67]. To counteract the harmful effects of ROS, plants possess various enzymatic and non-enzymatic defensive systems. These systems are activated in response to stress to prevent the excessive formation of free radicals and repair the damage caused by them [68,69]. IAA treatment increased total protein contents in mung and maize plants under individual, as well as combined stress conditions of Pb and NaCl [70,71], while SL3 treatment did not have a consistent effect on total protein content in the plants studied, except for a slight increase in maize plants under control conditions. Our results cooperate with those of [72] in that the IAA and SL3 treatment did not directly influence catalase activity under individual and combined stress of Pb and NaCl. Endophytic fungi are known to secrete phenolic compounds inside host tissues that cause a reduction in ROS concentration synthesized under various stresses [73]. In the current study, we have found that in mung plants, the total phenolic content tended to decrease under control conditions. However, the inoculation of SL3 increased the phenolic content under NaCl and combined NaCl and Pb stress conditions. In maize plants, the total phenolic contents were generally enhanced under stress conditions, with the highest levels observed in SL3-inoculated plants under combined NaCl and Pb stress. SL3 inoculation and exogenous treatment of IAA may have contrasting effects on PPO and flavanol activities under control conditions. However, in stress conditions of both Pb and NaCl, both IAA and SL3 positively influence the PPO and flavanol levels in the tested plants. These findings are in accordance with those found in the existing literature that an endophytic bacterial strain *Bacillus subtilis*, [74] significantly enhances antioxidant activities, including PPO and peroxidase (POX), that alleviate the oxidative stress effects resulting from salt and heavy metals in all treated plants compared to non-treated plants. SL3 has shown different effects on growth attributes and biochemical activities of mung and maize plants. These effects could be attributed to various factors, such as variations in their genetic makeup, metabolic pathways, and the specific mechanisms through which SL3 interacts with their physiological processes [75,76,77].

## 5. Conclusions

The findings of the current study indicate that the endophytic SL3 strain has the ability to alleviate salt and heavy metal stress in mung bean and maize plants by positively impacting various physiological and biochemical parameters. Overall, the study suggests that the utilization of the SL3 fungal strain as an IAA replacement offers an eco-friendly and cost-effective approach to enhance sustainable agriculture production in areas affected by salt and heavy metal stresses. However, further research is necessary to fully understand how SL3 influences the development and growth of plants under conditions of salt and heavy metal stress. 

## Data Availability

Not applicable.

## References

[1] Khan I., Khan S., Zhang Y., Zhou J., Akhoundian M., Jan S.A. (2021). CRISPR-Cas technology based genome editing for modification of salinity stress tolerance responses in rice (*Oryza sativa* L.). Mol. Biol. Rep..

[2] Khan I., Zhang Y., Akbar F., Khan J., Roychoudhury A., Aftab T., Acharya K. (2022). Abiotic Stress Tolerance in Cereals Through Genome Editing. Omics Approach to Manage Abiotic Stress in Cereals.

[3] Afzal M., Hindawi S.E.S., Alghamdi S.S., Migdadi H.H., Khan M.A., Hasnain M.U., Arslan M., Habib ur Rahman M., Sohaib M. (2023). Potential Breeding Strategies for Improving Salt Tolerance in Crop Plants. J. Plant Growth Regul..

[4] Zia R., Nawaz M.S., Siddique M.J., Hakim S., Imran A. (2021). Plant survival under drought stress: Implications, adaptive responses, and integrated rhizosphere management strategy for stress mitigation. Microbiol. Res..

[5] Lubna, Asaf S., Hamayun M., Khan A.L., Waqas M., Khan M.A., Jan R., Lee I.-J., Hussain A. (2018). Salt tolerance of *Glycine max* L. induced by endophytic fungus Aspergillus flavus CSH1, via regulating its endogenous hormones and antioxidative system. Plant Physiol. Biochem..

[6] Jhuma T.A., Rafeya J., Sultana S., Rahman M.T., Karim M.M. (2021). Isolation of Endophytic Salt-Tolerant Plant Growth-Promoting Rhizobacteria from *Oryza sativa* and Evaluation of Their Plant Growth-Promoting Traits under Salinity Stress Condition. Front. Sustain. Food Syst..

[7] Waqas M., Khan A.L., Kamran M., Hamayun M., Kang S.-M., Kim Y.-H., Lee I.-J. (2012). Endophytic Fungi Produce Gibberellins and Indoleacetic Acid and Promotes Host-Plant Growth during Stress. Molecules.

[8] Kruasuwan W., Lohmaneeratana K., Munnoch J.T., Vongsangnak W., Jantrasuriyarat C., Hoskisson P.A., Thamchaipenet A. (2023). Transcriptome Landscapes of Salt-Susceptible Rice Cultivar IR29 Associated with a Plant Growth Promoting Endophytic Streptomyces. Rice.

[9] Khan A.L., Halo B.A., Elyassi A., Ali S., Al-Hosni K., Hussain J., Al-Harrasi A., Lee I.-J. (2016). Indole acetic acid and ACC deaminase from endophytic bacteria improves the growth of Solanum lycopersicum. Electron. J. Biotechnol..

[10] Dubey A., Malla M.A., Kumar A., Dayanandan S., Khan M.L. (2020). Plants endophytes: Unveiling hidden agenda for bioprospecting toward sustainable agriculture. Crit. Rev. Biotechnol..

[11] Ur Rahman S., Li Y., Hussain S., Hussain B., Khan W.-u.-D., Riaz L., Nadeem Ashraf M., Athar Khaliq M., Du Z., Cheng H. (2023). Role of phytohormones in heavy metal tolerance in plants: A review. Ecol. Indic..

[12] Sytar O., Kumari P., Yadav S., Brestic M., Rastogi A. (2019). Phytohormone Priming: Regulator for Heavy Metal Stress in Plants. J. Plant Growth Regul..

[13] Bianco C., Defez R. (2009). Medicago truncatula improves salt tolerance when nodulated by an indole-3-acetic acid-overproducing Sinorhizobium meliloti strain. J. Exp. Bot..

[14] Kahraman A., Adali M., Onder M., Koc N., Kaya C. (2014). Mung bean [*Vigna radiata* (L.) Wilczek] as human food. Int. J. Agric. Econ. Dev..

[15] HanumanthaRao B., Nair R.M., Nayyar H. (2016). Salinity and high temperature tolerance in mungbean [*Vigna radiata* (L.) Wilczek] from a physiological perspective. Front. Plant Sci..

[16] Dahiya P., Linnemann A., Van Boekel M., Khetarpaul N., Grewal R., Nout M. (2015). Mung bean: Technological and nutritional potential. Crit. Rev. Food Sci. Nutr..

[17] Chaudhary D.P., Kumar S., Yadav O.P., Chaudhary D.P., Kumar S., Langyan S. (2014). Nutritive Value of Maize: Improvements, Applications and Constraints. Maize: Nutrition Dynamics and Novel Uses.

[18] Tanumihardjo S.A., McCulley L., Roh R., Lopez-Ridaura S., Palacios-Rojas N., Gunaratna N.S. (2020). Maize agro-food systems to ensure food and nutrition security in reference to the Sustainable Development Goals. Glob. Food Secur..

[19] Acosta J.A., Jansen B., Kalbitz K., Faz A., Martínez-Martínez S. (2011). Salinity increases mobility of heavy metals in soils. Chemosphere.

[20] Lacava P.T., Bogas A.C., Cruz F.D.P.N. (2022). Plant Growth Promotion and Biocontrol by Endophytic and Rhizospheric Microorganisms from the Tropics: A Review and Perspectives. Front. Sustain. Food Syst..

[21] Gang S., Sharma S., Saraf M., Buck M., Schumacher J. (2019). Analysis of Indole-3-acetic Acid (IAA) Production in Klebsiellaby LC-MS/MS and the Salkowski Method. Bio-Protocol.

[22] Lubna, Asaf S., Hamayun M., Gul H., Lee I.-J., Hussain A. (2018). Aspergillus niger CSR3 regulates plant endogenous hormones and secondary metabolites by producing gibberellins and indoleacetic acid. J. Plant Interact..

[23] Ullah I., Khan A.R., Park G.-S., Lim J.-H., Waqas M., Lee I.-J., Shin J.-H. (2013). Analysis of phytohormones and phosphate solubilization in *Photorhabdus* spp. Food Sci. Biotechnol..

[24] Vellore J.M. (2001). Iron Acquisition in Rhodococcus Erythrolpolis: The Isolation of Mutant (s) that Do Not Produce a Siderophore.

[25] Wildermuth E., Stark H., Friedrich G., Ebenhöch F.L., Kühborth B., Silver J., Rituper R. (2000). Iron compounds. Ullmann’s Encyclopedia of Industrial Chemistry.

[26] Novopol’tseva V.M., Labzina L., Atianina T.F. (1991). The preparation of iron (III) chloride solution from salt with an unknown water content. Lab. Delo.

[27] Lee I.-J., Foster K.R., Morgan P.W. (1998). Photoperiod control of gibberellin levels and flowering in sorghum. Plant Physiol..

[28] Khalmuratova I., Choi D.-H., Woo J.-R., Jeong M.-J., Oh Y., Kim Y.-G., Lee I.-J., Choo Y.-S., Kim J.-G. (2020). Diversity and plant growth-promoting effects of fungal endophytes isolated from salt-tolerant plants. J. Microbiol. Biotechnol..

[29] Barnes J.D., Balaguer L., Manrique E., Elvira S., Davison A.W. (1992). A reappraisal of the use of DMSO for the extraction and determination of chlorophylls a and b in lichens and higher plants. Environ. Exp. Bot..

[30] Bradford M.M. (1976). A rapid and sensitive method for the quantitation of microgram quantities of protein utilizing the principle of protein-dye binding. Anal. Biochem..

[31] Arulsekar S., Parfitt D.E. (1986). Isozyme Analysis Procedures for Stone Fruits, Almond, Grape, Walnut, Pistachio, and Fig. HortScience.

[32] Sánchez-Rangel J.C., Benavides J., Heredia J.B., Cisneros-Zevallos L., Jacobo-Velázquez D.A. (2013). The Folin–Ciocalteu assay revisited: Improvement of its specificity for total phenolic content determination. Anal. Methods.

[33] Illera A., Sanz M., Beltrán S., Melgosa R., Solaesa A., Ruiz M. (2018). Evaluation of HPCD batch treatments on enzyme inactivation kinetics and selected quality characteristics of cloudy juice from Golden delicious apples. J. Food Eng..

[34] Park Y.-S., Jung S.-T., Kang S.-G., Heo B.G., Arancibia-Avila P., Toledo F., Drzewiecki J., Namiesnik J., Gorinstein S. (2008). Antioxidants and proteins in ethylene-treated kiwifruits. Food Chem..

[35] Shin S.H., Lim Y., Lee S.E., Yang N.W., Rhee J.H. (2001). CAS agar diffusion assay for the measurement of siderophores in biological fluids. J. Microbiol. Methods.

[36] Shahid M.A., Sarkhosh A., Khan N., Balal R.M., Ali S., Rossi L., Gómez C., Mattson N., Nasim W., Garcia-Sanchez F. (2020). Insights into the physiological and biochemical impacts of salt stress on plant growth and development. Agronomy.

[37] Zhu J.-K. (2002). Salt and drought stress signal transduction in plants. Annu. Rev. Plant Biol..

[38] Gupta A., Mishra R., Rai S., Bano A., Pathak N., Fujita M., Kumar M., Hasanuzzaman M. (2022). Mechanistic insights of plant growth promoting bacteria mediated drought and salt stress tolerance in plants for sustainable agriculture. Int. J. Mol. Sci..

[39] Zhao S., Zhang Q., Liu M., Zhou H., Ma C., Wang P. (2021). Regulation of Plant Responses to Salt Stress. Int. J. Mol. Sci..

[40] Zhao C., Zhang H., Song C., Zhu J.-K., Shabala S. (2020). Mechanisms of Plant Responses and Adaptation to Soil Salinity. Innovation.

[41] Kratovalieva S., Cvetanowska L. (2001). Influence of different lead concentrations to some morpho-physiological parameters at tomato (*Lycopersicon esculentum* Mill.) in experimental conditions. Maced Agric. Rev..

[42] Angelova V.R., Babrikov T.D., Ivanov K.I. (2009). Bioaccumulation and distribution of lead, zinc, and cadmium in crops of Solanaceae family. Commun. Soil Sci. Plant Anal..

[43] Khan I., Asaf S., Jan R., Bilal S., Lubna, Khan A.L., Kim K.-M., Al-Harrasi A. (2023). Genome-wide annotation and expression analysis of WRKY and bHLH transcriptional factor families reveal their involvement under cadmium stress in tomato (*Solanum lycopersicum* L.). Front. Plant Sci..

[44] Finnegan P.M., Chen W. (2012). Arsenic toxicity: The effects on plant metabolism. Front. Physiol..

[45] Shahid M., Pourrut B., Dumat C., Nadeem M., Aslam M., Pinelli E., Whitacre D.M. (2014). Heavy-Metal-Induced Reactive Oxygen Species: Phytotoxicity and Physicochemical Changes in Plants. Reviews of Environmental Contamination and Toxicology.

[46] Ashraf M., Akram N.A. (2009). Improving salinity tolerance of plants through conventional breeding and genetic engineering: An analytical comparison. Biotechnol. Adv..

[47] Kumar A., Verma J.P. (2018). Does plant—Microbe interaction confer stress tolerance in plants: A review?. Microbiol. Res..

[48] Aizaz M., Ahmad W., Asaf S., Khan I., Saad Jan S., Salim Alamri S., Bilal S., Jan R., Kim K.-M., Al-Harrasi A. (2023). Characterization of the Seed Biopriming, Plant Growth-Promoting and Salinity-Ameliorating Potential of Halophilic Fungi Isolated from Hypersaline Habitats. Int. J. Mol. Sci..

[49] Gong Q., Li Z., Wang L., Dai T., Kang Q., Niu D. (2019). Exogenous of Indole-3-Acetic Acid Application Alleviates Copper Toxicity in Spinach Seedlings by Enhancing Antioxidant Systems and Nitrogen Metabolism. Toxics.

[50] Arif Y., Singh P., Siddiqui H., Bajguz A., Hayat S. (2020). Salinity induced physiological and biochemical changes in plants: An omic approach towards salt stress tolerance. Plant Physiol. Biochem..

[51] Ma C., Yuan S., Xie B., Li Q., Wang Q., Shao M. (2022). IAA Plays an Important Role in Alkaline Stress Tolerance by Modulating Root Development and ROS Detoxifying Systems in Rice Plants. Int. J. Mol. Sci..

[52] Ramadoss D., Lakkineni V.K., Bose P., Ali S., Annapurna K. (2013). Mitigation of salt stress in wheat seedlings by halotolerant bacteria isolated from saline habitats. SpringerPlus.

[53] Kumar V., Raghuvanshi N., Pandey A.K., Kumar A., Thoday-Kennedy E., Kant S. (2023). Role of halotolerant plant growth-promoting rhizobacteria in mitigating salinity stress: Recent advances and possibilities. Agriculture.

[54] Saleem S., Iqbal A., Ahmed F., Ahmad M. (2021). Phytobeneficial and salt stress mitigating efficacy of IAA producing salt tolerant strains in *Gossypium hirsutum*. Saudi J. Biol. Sci..

[55] Egamberdieva D., Jabborova D., Hashem A. (2015). Pseudomonas induces salinity tolerance in cotton (*Gossypium hirsutum*) and resistance to Fusarium root rot through the modulation of indole-3-acetic acid. Saudi J. Biol. Sci..

[56] Shi Y., Lou K., Li C. (2010). Growth and photosynthetic efficiency promotion of sugar beet (Beta vulgaris L.) by endophytic bacteria. Photosynth. Res..

[57] Li X., Bu N., Li Y., Ma L., Xin S., Zhang L. (2012). Growth, photosynthesis and antioxidant responses of endophyte infected and non-infected rice under lead stress conditions. J. Hazard. Mater..

[58] Ali B., Wang X., Saleem M.H., Azeem M.A., Afridi M.S., Nadeem M., Ghazal M., Batool T., Qayyum A., Alatawi A. (2022). Bacillus mycoides PM35 Reinforces Photosynthetic Efficiency, Antioxidant Defense, Expression of Stress-Responsive Genes, and Ameliorates the Effects of Salinity Stress in Maize. Life.

[59] Maslennikova D., Koryakov I., Yuldashev R., Avtushenko I., Yakupova A., Lastochkina O. (2023). Endophytic Plant Growth-Promoting Bacterium Bacillus subtilis Reduces the Toxic Effect of Cadmium on Wheat Plants. Microorganisms.

[60] Ikram M., Ali N., Jan G., Jan F.G., Rahman I.U., Iqbal A., Hamayun M. (2018). IAA producing fungal endophyte Penicillium roqueforti Thom., enhances stress tolerance and nutrients uptake in wheat plants grown on heavy metal contaminated soils. PLoS ONE.

[61] Tufail M.A., Bejarano A., Shakoor A., Naeem A., Arif M.S., Dar A.A., Farooq T.H., Pertot I., Puopolo G. (2021). Can Bacterial Endophytes Be Used as a Promising Bio-Inoculant for the Mitigation of Salinity Stress in Crop Plants?—A Global Meta-Analysis of the Last Decade (2011–2020). Microorganisms.

[62] Almuhayawi M.S., Abdel-Mawgoud M., Al Jaouni S.K., Almuhayawi S.M., Alruhaili M.H., Selim S., AbdElgawad H. (2021). Bacterial Endophytes as a Promising Approach to Enhance the Growth and Accumulation of Bioactive Metabolites of Three Species of Chenopodium Sprouts. Plants.

[63] Lushchak V.I. (2014). Free radicals, reactive oxygen species, oxidative stress and its classification. Chem.-Biol. Interact..

[64] Nita M., Grzybowski A. (2016). The role of the reactive oxygen species and oxidative stress in the pathomechanism of the age-related ocular diseases and other pathologies of the anterior and posterior eye segments in adults. Oxidative Med. Cell. Longev..

[65] Schieber M., Chandel N.S. (2014). ROS function in redox signaling and oxidative stress. Curr. Biol..

[66] Auten R.L., Davis J.M. (2009). Oxygen Toxicity and Reactive Oxygen Species: The Devil Is in the Details. Pediatr. Res..

[67] Hasanuzzaman M., Bhuyan M.H.M.B., Zulfiqar F., Raza A., Mohsin S.M., Mahmud J.A., Fujita M., Fotopoulos V. (2020). Reactive Oxygen Species and Antioxidant Defense in Plants under Abiotic Stress: Revisiting the Crucial Role of a Universal Defense Regulator. Antioxidants.

[68] Ahmad P., Jaleel C.A., Azooz M., Nabi G. (2009). Generation of ROS and non-enzymatic antioxidants during abiotic stress in plants. Bot. Res. Int..

[69] Sarker U., Oba S. (2018). Drought stress effects on growth, ROS markers, compatible solutes, phenolics, flavonoids, and antioxidant activity in Amaranthus tricolor. Appl. Biochem. Biotechnol..

[70] Hagaggi N.S.A., Mohamed A.A.A. (2020). Enhancement of *Zea mays* (L.) growth performance using indole acetic acid producing endophyte Mixta theicola isolated from Solenostemma argel (Hayne). South Afr. J. Bot..

[71] Abd El-Samad H.M. (2013). The physiological response of wheat plants to exogenous application of gibberellic acid (GA3) or indole-3-acetic acid (IAA) with endogenous ethylene under salt stress conditions. Int. J. Plant Physiol. Biochem..

[72] Kaya C., Ashraf M., Dikilitas M., Tuna A.L. (2013). Alleviation of salt stress-induced adverse effects on maize plants by exogenous application of indoleacetic acid (IAA) and inorganic nutrients-A field trial. Aust. J. Crop Sci..

[73] Hussain A., Ismail A., Qadir M., Hamayun M., Mehmood A. (2020). Thermal stress alleviating potential of endophytic fungus rhizopus oryzae inoculated to sunflower (*Helianthus annuus* L.) and soybean (*Glycine max* L.). Pak. J. Bot..

[74] El-Gendi H., Al-Askar A.A., Király L., Samy M.A., Moawad H., Abdelkhalek A. (2022). Foliar Applications of Bacillus subtilis HA1 Culture Filtrate Enhance Tomato Growth and Induce Systemic Resistance against Tobacco mosaic virus Infection. Horticulturae.

[75] Khan A.L., Hussain J., Al-Harrasi A., Al-Rawahi A., Lee I.-J. (2015). Endophytic fungi: Resource for gibberellins and crop abiotic stress resistance. Crit. Rev. Biotechnol..

[76] Gupta A., Singh A.N., Tiwari R.K., Sahu P.K., Yadav J., Srivastava A.K., Kumar S. (2023). Salinity Alleviation and Reduction in Oxidative Stress by Endophytic and Rhizospheric Microbes in Two Rice Cultivars. Plants.

[77] Poveda J., Baptista P., Sacristán S., Velasco P. (2022). Editorial: Beneficial effects of fungal endophytes in major agricultural crops. Front. Plant Sci..

